# The Efficacy and Safety of Eravacycline in the Management of Infections: A Single-Center Experience

**DOI:** 10.3390/antibiotics15050474

**Published:** 2026-05-07

**Authors:** Narendran Koomanan, Yvonne Peijun Zhou, Andrea Lay Hoon Kwa

**Affiliations:** 1 Division of Pharmacy, Singapore General Hospital, Outram Road, Singapore 169608, Singapore; narendran.koomanan@sgh.com.sg (N.K.); yvonne.zhou.p.j@sgh.com.sg (Y.P.Z.); 2 SingHealth Duke-NUS Medicine Academic Clinical Programme, 8 College Road, Singapore 169857, Singapore; 3 Emerging Infectious Disease Program, Duke-NUS Medical School, 8 College Road, Singapore 169857, Singapore

**Keywords:** eravacycline, multidrug-resistant organisms (MDROs), nontuberculous mycobacteria (NTM), Desirability of Outcome Ranking (DOOR) framework

## Abstract

**Background/Objectives**: Eravacycline is a fluorocycline antibiotic increasingly used for drug-resistant or difficult-to-treat infections, including off-label indications, with limited real-world clinical data. This study aimed to characterize the effectiveness, safety, and overall risk-benefit profile of eravacycline using an adapted Desirability of Outcome Ranking (DOOR) framework. **Methods**: We conducted a retrospective, single-center observational study of adult patients who received ≥48 h of eravacycline at an academic medical center between May 2022 and October 2023. Clinical response was assessed at the end of therapy, alongside 30-day all-cause mortality. Treatment-emergent adverse events (TEAEs) were recorded and normalized per 100 eravacycline-days. An adapted DOOR framework integrated efficacy, toxicity and mortality into an ordinal composite outcome, with analyses stratified by pathogen and site of infection. **Results**: A total of 140 patients contributed 151 eravacycline courses. Intra-abdominal (41.7%) and lower respiratory tract infections (27.8%) were the most common indications. Treatment success was observed in 69.5% of courses, while 30-day all-cause mortality was 23.6%. TEAEs occurred in 52.3% of courses and frequently led to eravacycline discontinuation. Exposure-normalized TEAE rates were highest in shorter courses, with gastrointestinal intolerance predominating early, while hepatoxicity and coagulation abnormalities were more frequent with intermediate treatment durations. DOOR analysis demonstrated highly desirable outcomes in 48.3% of courses, with more favorable profiles observed in carbapenem-resistant *Enterobacterales* (CRE), vancomycin-resistant *Enterococci* (VRE) and *nontuberculous mycobacteria* (NTM) infections. Bloodstream infections were associated with less desirable outcomes. **Conclusions**: Eravacycline demonstrated meaningful real-world activity across complex infections but was limited by frequent toxicity. The DOOR framework provided a patient-centered context for organism- and site-specific risk-benefit assessment.

## 1. Introduction

Driven by inappropriate and excessive use of anti-infectives, the global burden of antimicrobial resistance (AMR) continues to rise, posing a serious threat to public health [1,2]. While the development of new antibiotics has slowed considerably, recently approved agents have offered limited but important advances in the treatment of resistant infections [3]. Among these is eravacycline, a novel fluorocycline antibiotic approved by the U.S. Food and Drug Administration (FDA) for the treatment of complicated intra-abdominal infections in adults.

Eravacycline exhibits broad-spectrum activity against Gram-positive, Gram-negative, anaerobic, and atypical pathogens. In vitro studies have demonstrated potent activity against multidrug-resistant organisms (MDROs), including *Acinetobacter baumannii*, *Stenotrophomonas maltophilia*, and *Enterobacterales* producing Class A, B, and D carbapenemases [4,5,6,7,8,9]. Studies have also suggested that eravacycline might be a useful component of treatment regimens for *nontuberculous mycobacteria* (NTM) infections [9]. These properties have led to increasing off-label use of eravacycline in clinical practice for the treatment of difficult-to-treat or drug-resistant infections.

However, despite encouraging in vitro data, clinical evidence supporting the safety and effectiveness of eravacycline in real-world settings, particularly for off-label indications, remains limited [10,11]. Real-world use is often characterized by prolonged durations, high comorbidity burden, and frequent use of concurrent antimicrobials, complicating assessment of efficacy and safety. Conventional analyses report these outcomes separately, and as such, fail to fully capture the overall risk-benefit trade-offs inherent in these complex clinical scenarios.

The Desirability of Outcome Ranking (DOOR) framework has been proposed as a patient-centered approach to integrate efficacy, safety and mortality into a single ordinal outcome, allowing a more holistic assessment of therapeutic impact. DOOR has been increasingly applied in antimicrobial studies to contextualize trade-offs between clinical response and adverse events [12]. However, its application in single-arm observational studies remains largely descriptive and does not permit direct comparative influence.

In this study, we describe the real-world use of eravacycline in a single-center cohort for both labeled and off-label indications. We aimed to evaluate the overall risk-benefit profile of eravacycline using an adapted DOOR framework.

## 2. Results

Of the 159 patients prescribed eravacycline, 17 received <48 h of therapy and two did not receive any doses; these patients were excluded. The remaining 140 patients contributed 151 treatment courses that met the inclusion criteria. Five courses belonging to the same infectious episode as a prior course were excluded. Eight patients received multiple courses of eravacycline (19 courses in total), all initiated for new septic events occurring ≥1 week after completion of the previous eravacycline course. The mean (SD) patient age was 61.2 (15) years, with 52.1% males. The median (IQR) Charlson Comorbidity Index was 5 (3–7), and 23.6% of patients were immunocompromised (Table 1).

Eravacycline was most frequently used for intra-abdominal infections (41.7%), followed by lower respiratory tract infections (27.8%); of which 19/42 (45.2%) were for NTM infections. Source control interventions were performed in 33.1% of courses, and 21.9% required admission to the intensive care unit (Table 2).

Polymicrobial infections were present in 39.7% of the courses and the isolated organisms are summarized in Table 3. Specimen sources from which pathogens were isolated are summarized in Appendix A Table A1. Eravacycline was often used to cover *Enterobacterales* (60/253, 23.7%), with 35% (21/60) being carbapenem-resistant. *Enterococci* species were also commonly isolated (54/253, 21.3%), with 38.9% (21/54) being resistant to vancomycin. Eravacycline was used for the treatment of *Acinetobacter baumannii* infections (22/253, 8.7%), the vast majority of which were carbapenem-resistant (20/22, 90.1%). Eravacycline also served as a treatment option for NTM (26/253, 10.3%) and *Stenotrophomonas maltophilia* infections (17/253, 6.7%). Approximately two-thirds of courses were pathogen-directed, including seven guided by in-house multiple combination bactericidal testing (MCBT).

Beyond microbiological considerations, eravacycline selection was driven by the need to consolidate antibiotic therapy (27.2%) and mitigate adverse drug events (27.2%). Its broad antimicrobial spectrum and suitability for outpatient antimicrobial therapy (OPAT) were also key considerations (Table 4). Standard dosing (1 mg/kg Q12H) was used in 73.5% of the courses, with modified dosing employed to facilitate OPAT or mitigate toxicity. The median treatment duration was 14 (IQR: 8–27) days. Eravacycline was commonly co-administered with other antibiotics, most frequently quinolones (18.5%) and carbapenems (17.2%), as detailed in Table 4. The reported duration reflects eravacycline exposure only and does not represent the total duration of antimicrobial therapy, which might include prior or subsequent agents or was limited by TEAEs.

Treatment success was observed in 69.5% of eravacycline treatment courses, with 21.2% achieving complete clinical cure (Table 5). Among treatment failures, common contributing factors were the emergence of new infectious complications (19/46, 41.3%) and clinical deterioration or breakthrough infections (18/46, 39.1%). Mortality occurred in 23.6% (33/140) of patients, with 13 events attributed to infection-related causes.

Treatment-emergent adverse events (TEAEs) occurred in 52.3% of eravacycline treatment courses. When normalized to exposure duration, the overall TEAE rate was 2.84 events per 100 eravacycline-days. TEAE rates were highest among shorter courses and decreased with longer treatment duration, with rates of 9.43 events per 100 eravacycline-days for courses lasting ≤7 days, 6.11 events per 100 eravacycline-days for courses lasting 8–14 days, and 1.63 events per 100 eravacycline-days for courses lasting ≥15 days (Table A2). The distribution of adverse event types varied by treatment duration. Gastrointestinal adverse events were more prevalent among shorter courses (≤7 days), whereas hepatotoxicity and elevated aPTT were more frequently observed among intermediate treatment courses (8–14 days). These adverse events led to discontinuation of eravacycline in a substantial proportion of affected courses (Table 5). Among courses complicated by elevated aPTT, coagulation parameters generally improved within one week of discontinuation, whereas normalization of liver function tests was more protracted.

**Figure 1:**
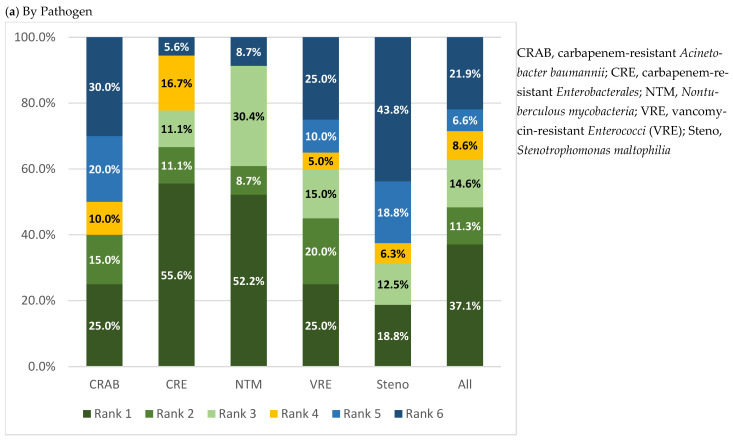

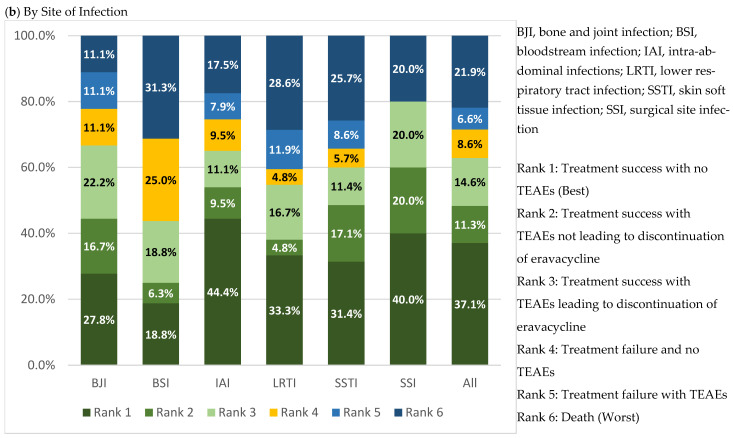
Cumulative Distribution of DOOR ranks for Eravacycline Treatment Courses.

Using the DOOR framework, 48.3% of courses achieved highly desirable outcomes (Rank 1–2), while 21.9% fell into the least desirable category (Rank 6). The distribution of DOOR ranks by pathogen and the site of infection are summarized in Figure 1. Stratification by pathogen demonstrated more desirable outcomes among courses treating CRE, VRE and NTM infections, whereas less desirable outcomes were more frequently observed among courses treating CRAB and *Stenotrophomonas maltophilia* infections. When stratified by site of infection, high proportions of desirable outcomes were observed in intra-abdominal infections, surgical site infections and skin and soft tissue infections, while bloodstream infections had the least desirable outcomes (Figure 1 and Table A3 and Table A4).

## 3. Discussion

This study describes the real-world use of eravacycline following its inclusion in our institutional formulary and provides a descriptive assessment of clinical response, safety, and overall risk-benefit across a heterogenous population. While intra-abdominal infections were the most common indication, eravacycline was frequently used for off-label indications, including pneumonia and skin and soft tissue infections. Its broad-spectrum activity made it a preferred option for streamlining antimicrobial therapy and mitigating the risk of toxicity associated with alternative agents. In this cohort, treatment success was observed in 69.5% of eravacycline treatment courses, comparable to response rates reported in other real-world studies [13]. However, TEAEs were common and influenced treatment continuation.

Clinical outcomes varied substantially by pathogen. Favorable responses were observed among courses targeting CRE and VRE, supporting the latest IDSA guidance that recognizes eravacycline as an alternative option for selected CRE infections [14]. In contrast, outcomes for CRAB were less favorable, with fewer than half of the courses achieving treatment success. Treatment failure in CRAB infections frequently occurred with new septic events or breakthrough bacteremia, underscoring the need to further evaluate the role of eravacycline in the management of MDROs and suggesting that its effectiveness may be contingent on organism-specific factors, source control, and combination strategies. These findings align with existing literature that highlights uncertainty regarding the optimal role of eravacycline in CRAB infections [13,14,15,16,17].

Clinical experience with eravacycline for *nontuberculous mycobacteria* (NTM) is scarce, despite encouraging in vitro data [6,8,9]. In our cohort, most NTM treatment courses had clinical improvement. Treatment success was still observed even when therapy was discontinued early, or the dose was adjusted due to adverse events. While these observations suggest the potential efficacy of eravacycline in NTM therapy, interpretation is limited by small sample size, concomitant multi-drug regimens, and the complexity of NTM treatment. Further prospective evaluation is needed before routine use can be recommended in this setting.

The duration of eravacycline therapy in this cohort reflects its role within broader antimicrobial regimens rather than total treatment duration, with early discontinuation or dose adjustment frequently influenced by TEAEs.

The incidence of TEAEs in our cohort exceeds rates reported in prior clinical trials and observational studies [13,17,18,19,20,21,22]. Several factors may have contributed to this finding. First, treatment duration was longer in our population, with a median duration of 14 days compared to 7–8 days in previous reports. Second, patients had a high burden of comorbidities and extensive exposure to concomitant antimicrobials, complicating the attribution of adverse events to eravacycline use alone. When adverse events were normalized to eravacycline exposure, higher rates were observed in shorter treatment courses, with progressively lower rates in longer courses. However, the type of adverse events varied by treatment duration. Gastrointestinal intolerance predominated early in therapy and likely contributed to early discontinuation in susceptible patients. In contrast, hepatotoxicity and coagulation abnormalities were more frequently observed after intermediate durations of exposure (8–14 days), suggesting these adverse events may be cumulative or delayed in onset. This pattern may explain the apparent decline in overall adverse event rates with longer treatment duration, reflecting early intolerance and depletion of susceptible individuals rather than reduced cumulative toxicity with prolonged exposure. Elevated aPTT was the most frequently observed adverse event and warrants closer monitoring, particularly during prolonged therapy, given limited prior reporting and uncertain clinical significance [16]. In our cohort, coagulation abnormalities generally improved following treatment discontinuation.

To complement conventional endpoints, an adapted DOOR framework was applied to integrate clinical response, toxicity, and mortality into a single, patient-centered assessment of overall treatment desirability. Nearly half of the treatment courses achieved highly desirable outcomes (Ranks 1,2), while approximately one-fifth were classified as least desirable (Rank 6). When pathogen-specific clinical responses were contextualized using the DOOR framework, more favorable risk-benefit profiles were observed among courses treating CRE, NTM, and VRE infections, while less desirable outcomes were more common for courses treating CRAB or *Stenotrophomonas maltophilia*. These findings highlight the organism-specific challenges observed with eravacycline use and may help inform clinical decision-making. Stratification by site of infection further highlighted important differences in overall outcome desirability. Courses treating intra-abdominal infections, surgical site infections, and skin and soft tissue infections were more frequently associated with desirable DOOR ranks, whereas bloodstream infections were associated with the least desirable outcomes. This likely reflects both disease severity and pharmacokinetic considerations, including relatively low serum concentrations of eravacycline, which may limit its effectiveness in bloodstream infections. Although this single-arm DOOR adaptation does not provide comparative probabilities versus alternative agents, it offers a pragmatic, patient-centered view on eravacycline’s risk-benefit profile across different clinical scenarios and may inform more selective, indication-specific use.

This study has several limitations. The retrospective, single-center design and the absence of a comparator arm limit causal inference and the ability to attribute outcomes specifically to eravacycline. Although the study included patients treated for MDROs and NTM infections, the small subgroup sizes limit the generalizability of the findings. Course-level analysis may overrepresent patients with multiple treatment episodes, and outcome classification remains subject to residual misclassification. Additionally, inclusion of all adverse events, regardless of their attribution, may overestimate the true incidence of eravacycline-related toxicity. For the DOOR adaptation, misclassification bias may arise from retrospective adjudication of clinical response and TEAE severity, and our single-arm framework precludes estimation of the probability that eravacycline yields a more desirable outcome compared to alternative therapies. Despite these limitations, this study provides valuable real-world data on eravacycline use across a broad range of difficult-to-treat infections and demonstrates the utility of DOOR as a pragmatic, patient-centered framework for contextualizing risk-benefit in a heterogeneous, high-risk population.

## 4. Materials and Methods

This was a retrospective, single-center observational study conducted at Singapore General Hospital (SGH), a 2000-bed academic medical center and tertiary referral hospital. We retrospectively reviewed the medical records of all adult patients who received at least 48 h of eravacycline between May 2022 and October 2023, for either empiric or definitive treatment of any infection. For definitive treatment, at least one isolated pathogen had to be susceptible to eravacycline.

Patients were excluded if they received less than 48 h of eravacycline or were expected to die within 48 h of treatment initiation. For patients with multiple admissions during the study period, only admissions occurring at least 90 days apart were included. For patients who received multiple courses of eravacycline during a single admission, only courses initiated for a new infectious episode were counted as distinct courses. When multiple courses were given within the same infectious episode, only the first course was included in the analysis.

The primary outcome was clinical response at the end of a course of eravacycline, classified as: (1) clinical cure–defined as complete resolution of infection-related signs and symptoms with no requirement for further antimicrobial therapy; (2) clinical improvement–defined as partial resolution or stabilization of infection-related signs and symptoms accompanied by at least one of the following: (a) a consistent downward trend towards institutional reference ranges in infection-related biomarkers; (b) de-escalation of antimicrobial therapy; or (c) transition to oral therapy; (3) indeterminate response–defined as cases in which neither clinical improvement nor clinical failure could be confidently determined due to limited clinical documentation or absence of response indicators; (4) clinical failure–defined as persistence or worsening of infection-related signs and symptoms, escalation or change in antimicrobial therapy due to lack of efficacy. De-escalation or transition to oral therapy performed for non-clinical reasons, including adverse events, logistical considerations, or lack of intravenous access, was not considered evidence of clinical improvement. The clinical response reflected the response achieved during therapy and was assessed irrespective of subsequent mortality. Treatment success comprised clinical cure and improvement (categories 1 and 2), while treatment failure encompassed indeterminate response and clinical failure (categories 3 and 4). Secondary outcomes included 30-day all-cause mortality and 30-day infection-related mortality from initiation of eravacycline. The safety outcome was the incidence of treatment-emergent adverse events (TEAEs), including those leading to the discontinuation of eravacycline. TEAEs were additionally normalized and expressed as rates per 100 eravacycline-days. Rates were stratified by treatment duration (≤7 days, 8 to 14 days, and ≥15 days) to explore the relationship between exposure duration and adverse events.

To provide an integrated assessment of risk-benefit, a DOOR framework was applied as a secondary analytic approach. Each course of eravacycline was assigned a DOOR rank based on a composite of clinical response and TEAEs at the end of eravacycline therapy. Courses were assigned to one of six mutually exclusive ranks, with rank 1 representing the most desirable outcome and rank 6 representing the least desirable outcome. Rank 1 represented courses with treatment success and no TEAEs. Ranks 2 and 3 represented treatment success with TEAEs not leading to discontinuation (Rank 2) and TEAEs leading to discontinuation of eravacycline (Rank 3). Ranks 4 and 5 represented treatment failure without (Rank 4) and with TEAEs (Rank 5). Rank 6 represented all courses in which the patient died within 30 days of eravacycline initiation and superseded all other clinical and safety outcomes, regardless of interim clinical response. For courses with multiple adverse events, the most severe TEAEs were considered for DOOR classification. The distribution of DOOR ranks was summarized by pathogen and site of infection.

Patient demographics and baseline characteristics were extracted from electronic medical records. Comorbidity burden was estimated using the Charlson Comorbidity Index (CCI) and sepsis severity was estimated using the quick Sequential Organ Failure Assessment (qSOFA) score within 48 h of eravacycline initiation. Patients were considered immunosuppressed if they had active malignancy and were receiving chemotherapy, were on high-dose corticosteroids (at least prednisolone 15 mg/day for 14 days or its equivalent), were receiving immunosuppressive therapy, or had any other underlying immune deficiency. Microbiological data, including positive cultures closest to the initiation of eravacycline, were recorded. Treatment-related data such as rationale for choosing eravacycline, dose and duration, and use of concomitant antibiotics were collected. Concomitant therapy was defined as any antibiotic administered concurrently with eravacycline for the treatment of the primary infection.

Discrete data were summarized with frequencies and percentages, while continuous data were reported using mean and standard deviation or median and interquartile range. The distribution of DOOR ranks was presented as frequencies and percentages.

## 5. Conclusions

This study provides real-world insights into the use of eravacycline across a diverse range of infections, including off-label indications, NTM and MDROs. Clinical response rates were comparable to those reported in existing literature, although TEAEs were more frequent in this cohort, reflecting longer treatment durations, patient complexity, and frequent concomitant antimicrobial use. While overall adverse event rates were highest early in therapy, the type of toxicity varied with duration, with gastrointestinal intolerance predominating in shorter courses and hepatotoxicity and coagulation abnormalities emerging with intermediate exposure. The observed incidence of elevated aPTT warrants further investigation to clarify underlying mechanisms, risk factors and clinical significance.

Integration of the DOOR framework provided a patient-centered synthesis of efficacy, safety, and mortality, highlighting differences in outcomes by pathogen and site of infection. Eravacycline demonstrated more favorable risk-benefit profiles when used for CRE, VRE and NTM infections, whereas outcomes for CRAB were less favorable and frequently complicated by breakthrough infections. Stratification by site of infection further highlighted heterogeneity in outcomes, with more desirable DOORs observed in intra-abdominal, surgical site, and skin and soft tissue infections, and the least desirable outcomes observed in bloodstream infections.

Overall, integration of the DOOR framework provided a patient-centered synthesis of efficacy, safety, and mortality, highlighting the trade-offs inherent in treating high-risk infections and supporting a more selective, indication-specific role for eravacycline pending further comparative studies.

## Figures and Tables

**Table 1:** Baseline Demographics.

Patient Characteristics	Patients (n = 140)
Age, mean (SD)	61.2 (15)
- Male, n (%)	73 (52.1%)
- Female, n (%)	67 (47.9%)
Ethnicity, n (%)	
- Chinese	101 (72.1%)
- Malay	17 (12.2%)
- Indian	17 (12.2%)
- Others	5 (3.6%)
Weight (kg), median (IQR)	59.9 (49.2–69.3)
BMI (kg/m^2^), median (IQR)	22.8 (18.8–26.5)
Allergies, n (%)	
- Any beta-lactam allergy	25 (17.9%)
Co-morbidities, n (%)	
- Bronchiectasis/COPD	14 (10.0%)
- Diabetes	46 (32.9%)
- Kidney disease	32 (22.9%)
- Dialysis-dependent	21/32 (65.6%)
- Malignancy	60 (42.9%)
- Immunocompromised host ^a^	33 (23.6%)
Severity Scores, median (IQR)	
- Charlson Comorbidity Index	5 (3–7)
- qSOFA (on eravacycline initiation)	0 (0–1)
LOS (days), median (IQR)	46 (21.5–80.5)

^a^ Immunocompromised host includes patients with active malignancy receiving chemotherapy (n = 20), on high-dose corticosteroid therapy (n = 6), on other immunosuppressant therapy (n = 3), or with other underlying immune deficiency (n = 4). SD, standard deviation; IQR, interquartile range; COPD, chronic obstructive pulmonary disease; qSOFA, quick Sequential Organ Failure Assessment score; LOS, length of stay

**Table 2:** Infection-related Characteristics.

Infection-Related Characteristics	Eravacycline Courses, n (%) (n = 151)
Infection type ^a^	
- Bone and joint infection	18 (11.9%)
•NTM	1/18 (5.6%)
- Bloodstream infection	16 (10.6%)
- Intra-abdominal infection	63 (41.7%)
- Lower respiratory tract infection	42 (27.8%)
•NTM	19/42 (45.2%)
- Skin and soft tissue infection	35 (23.2%)
•NTM	3/35 (8.6%)
- Surgical site infection	10 (6.6%)
- Others ^b^	5 (3.3%)
Admission to intensive care unit	33 (21.9%)
Any surgical intervention	50 (33.1%)
Polymicrobial infections	60 (39.7%)

^a^ Patients might have more than 1 type of infection for each course; ^b^ Other infections included urinary tract infections (n = 3), reproductive tract infection (n = 1), and unspecified sepsis (n = 1); NTM, nontuberculous mycobacteria.

**Table 3:** Microbiological Characteristics.

Microbiological Characteristics			All Isolates (n = 253) ^a^	
Gram-negative				
	*Achromobacter* spp.		2	(0.8%)
	*Acinetobacter baumannii*		22	(8.7%)
		Carbapenem-resistant	20/22	(90.1%)
	*Enterobacterales*			
		*Citrobacter koseri*	1	(0.4%)
		*Cronobacter* spp.	1	(0.4%)
		*Enterobacter cloacae*	5	(2.0%)
		*Escherichia coli*	12	(4.7%)
		*Klebsiella pneumoniae*	12	(4.7%)
		*Morganella morganii*	1	(0.4%)
		*Proteus mirabilis*	7	(2.8%)
	Carbapenem-resistant *Enterobacterales*			
		*Citrobacter freundii*	1	(0.4%)
		*Enterobacter cloacae*	4	(1.6%)
		*Escherichia coli*	6	(2.4%)
		*Klebsiella pneumoniae*	10	(4.0%)
	*Pseudomonas aeruginosa*		14	(5.5%)
		Carbapenem-resistant	2/14	(14.3%)
	*Stenotrophomonas maltophilia*		17	(6.7%)
	*Aeromonas* spp.		2	(0.8%)
	*Elizabethkingia species*		1	(0.4%)
Gram-positive				
	*Enterococci*			
		*Enterococcus faecalis*	16	(6.3%)
		*Enterococcus faecium*	9	(3.6%)
		*Enterococcus raffinosus*	3	(1.2%)
		*Enterococcus gallinarum*	3	(1.2%)
		*Enterococcus avium*	2	(0.8%)
		Vancomycin-resistant *enterococci*	21	(8.3%)
	*Staphylococcus aureus*			
		MSSA	1	(0.4%)
		MRSA	6	(2.4%)
		Coagulase-negative *staphylococci*	7	(2.8%)
		*S. lugdunensis*	2	(0.8%)
*Mycobacterium* spp.				
		*Mycobacterium abscessus*	20	(7.9%)
		*Mycobacterium fortuitum*	5	(2.0%)
		*Mycobacterium chelonae*	1	(0.4%)
Anaerobes ^b^			6	(2.4%)
Others ^c^			11	(4.3%)

^a^ Total number of isolated pathogens exceeds number of eravacycline courses due to polymicrobial infections; ^b^ Anaerobes include *B. fragilis* (n = 2), *Clostridium* spp. (n = 3), and *D. hominis* (n = 1); ^c^ Other pathogens include *Ureaplasma* spp. (n = 1), *C. indologenes* (n = 1), *Corynebactrium straitum* (n = 3), *Shewanella putrefaciens* (n = 1), *Cutibacterium acnes* (n = 2), *Necropsobacter rosorum* (n = 1), *P. putida* group (n = 1), and *Ochribacterum intermedium* (n = 1); spp., species; MSSA, methicillin-susceptible *S. aureus*; MRSA, methicillin-resistant *S. aureus.*

**Table 4:** Eravacycline and treatment-related data.

Eravacycline and Treatment-Related Data	Eravacycline Courses, n (%) (n = 151)
Culture-directed therapy	99 (65.6%)
- MCBT-guided therapy	7/99 (7.1%)
Rationale for choosing eravacycline ^a^	
- Avoidance of adverse reaction	41 (27.2%)
- Consideration for outpatient parenteral antibiotic therapy	23 (15.2%)
- Consolidation of antibiotic therapy	41 (27.2%)
- Enhance antibiotic cover	32 (21.2%)
Eravacycline dose	
- 1 mg/kg Q12H	111 (73.5%)
- 1.5 mg/kg Q24H	8 (5.3%)
- 1 mg/kg Q12H, then 1.5 mg/kg Q12H	17 (11.3%)
- Other dosing regimens ^b^	15 (9.9%)
Eravacycline duration (days), median (IQR)	14 (8–27)
Concurrent antibiotic therapy ^c^	
- None	58 (38.4%)
- Aminoglycosides	13 (8.6%)
- Aztreonam	13 (8.6%)
- Carbapenems	26 (17.2%)
- Cefepime	4 (2.6%)
- Cefoxitin	13 (8.6%)
- Ceftazidime	6 (4.0%)
- Ceftazidime-avibactam	3 (2.0%)
- Ceftolozane-tazobactam	2 (1.3%)
- Clofazimine	6 (4.0%)
- Daptomycin	3 (2.0%)
- Linezolid or Tedizolid	5 (3.3%)
- Macrolides	16 (10.6%)
- Piperacillin-tazobactam	2 (1.3%)
- Polymyxins	12 (7.9%)
- Quinolones	28 (18.5%)
- Trimethoprim/Sulfamethoxazole	6 (4.0%)
- Vancomycin	3 (2.0%)
- Others ^d^	4 (2.6%)

^a^ There could be multiple reasons for selecting eravacycline for each treatment course. ^b^ Other dosing regimens included dose rounding to the nearest vial size (n = 10), dose based on incorrect weight (n = 2), lower dose [0.33 mg/kg Q8H] due to adverse event (n = 1), higher dose [1.5 mg/kg Q12H] due of drug–drug interaction with phenytoin, and reason unknown (n = 1); ^c^ For each eravacycline course, patients might have received multiple concomitant antibiotics. ^d^ Other antibiotics include fosfomycin (n = 3), rifampicin, and ethambutol (n = 1); MCBT, multiple combination bactericidal testing; NTM, nontuberculous mycobacteria; Q12H, dose every 12 h, Q24H; dosed every 24 h; IQR, interquartile range.

**Table 5:** Efficacy and Safety Outcomes.

Efficacy and Safety Outcomes			Eravacycline Courses, n (%) (n = 151)	
Efficacy Indictors				
	Treatment success			
		Clinical cure	32	(21.2%)
		Clinical improvement	73	(48.3%)
	Treatment failure			
		Indeterminate response	3	(2.0%)
		Clinical failure	43	(28.5%)
	30-day all-cause mortality ^a^		33/140	(23.6%)
		Mortality due to infections ^b^	13/33	(39.4%)
		Mortality due to other causes	20/33	(60.6%)
Safety Indicators ^c^				
	Presence of any treatment-emergent adverse events (TEAEs)		79	(52.3%)
		Elevated aPTT	39	(25.8%)
		Coagulopathy and/or bleeding manifestations	24/39	(61.5%)
		Gastrointestinal symptoms	18	(11.9%)
		Hepatotoxicity	33	(21.9%)
	Discontinuation of eravacycline due to TEAE			
		Coagulopathy and/or bleeding manifestations	14/39	(35.9%)
		Gastrointestinal symptoms	12/18	(66.7%)
		Hepatotoxicity	9/33	(27.3%)

^a^ Proportion calculated based on total number of included patients (n = 140); ^b^ Of the 13 infection-related deaths, 6 were considered possibly related to the infection treated with eravacycline; ^c^ For each eravacycline course, patients may have developed multiple treatment-emergent adverse events; aPPT, activated partial thromboplastin time.

## Data Availability

The raw data supporting the conclusions of this article will be made available by the authors on request.

## Appendix A. Culture Specimen of Isolated Pathogens

**Table A1:** Culture specimen of isolated pathogens.

Culture Specimen	CRAB (n = 20)	CRE (n = 18)	NTM (n = 23)	VRE (n = 20)	*S. maltophilia* (n = 16)	All Courses (n = 151)
Blood	6 (30%)	3 (17%)	0 (0%)	2 (10%)	2 (13%)	18 (12%)
Bone	1 (5%)	0 (0%)	0 (0%)	0 (0%)	1 (6%)	4 (3%)
Fluid	0 (0%)	9 (50%)	1 (4%)	9 (45%)	2 (13%)	30 (20%)
Respiratory culture	8 (40%)	0 (0%)	16 (70%)	2 (10%)	5 (31%)	32 (22%)
Tissue	8 (40%)	7 (39%)	2 (9%)	11 (55%)	6 (38%)	41 (27%)
Urine	0 (0%)	1 (6%)	0 (0%)	0 (0%)	0 (0%)	2 (13%)

n refers to the number of eravacycline treatment courses; CRAB, carbapenem-resistant *Acinetobacter baumannii*; CRE, carbapenem-resistant *Enterobacterales*; NTM, *Nontuberculous mycobacteria*; VRE, vancomycin-resistant *Enterococci* (VRE); *S. maltophilia*, *Stenotrophomonas maltophilia.*

## Appendix B. Normalized Treatment-Emergent Adverse Events (TEAEs) Rates

**Table A2:** Treatment-emergent adverse events (TEAEs) rate.

Characteristics	Total Eravacycline Exposure (Days)	Number of TEAE	Rate of TEAE (per 100 Eravacycline Days)
Eravacycline Treatment duration			
≤7 days	159	15	9.43
8 to 14 days	475	29	6.10
≥15 days	2149	35	1.62
All courses	2783	79	2.84
Concurrent antibiotic therapy			
Yes	1859	54	2.90
No	924	25	2.71

## Appendix C. Distribution of Desirability of Outcome Ranking (DOOR) Scores Among Eravacycline Treatment Courses

**Table A3:** By Pathogen.

Pathogen	Rank 1	Rank 2	Rank 3	Rank 4	Rank 5	Rank 6
Carbapenem-resistant *Acinetobacter baumannii* (CRAB)	5 (25%)	3 (15%)	0 (0%)	2 (10%)	4 (20%)	6 (30%)
Carbapenem-resistant *Enterobacterales* (CRE)	10 (56%)	2 (11%)	2 (11%)	3 (17%)	0 (0%)	1 (6%)
*Nontuberculous mycobacteria* (NTM)	12 (52%)	2 (9%)	7 (30%)	0 (0%)	0 (0%)	2 (9%)
Vancomycin-resistant *Enterococci* (VRE)	5 (25%)	4 (20%)	3 (15%)	1 (5%)	2 (10%)	5 (25%)
*Stenotrophomonas maltophilia*	3 (19%)	0 (0%)	2 (12%)	1 (6%)	3 (19%)	7 (44%)
All	56 (37%)	17 (11%)	22 (15%)	13 (9%)	10 (7%)	33 (22%)

**Table A4:** By Site of Infection.

Site of Infection	Rank 1	Rank 2	Rank 3	Rank 4	Rank 5	Rank 6
Bone and joint infection	5 (28%)	3 (17%)	4 (22%)	2 (11%)	2 (11%)	2 (11%)
Bloodstream infection	3 (19%)	1 (6%)	3 (19%)	4 (25%)	0 (0%)	5 (31%)
Intra-abdominal infections	28 (44%)	6 (10%)	7 (11%)	6 (10%)	5 (8%)	11 (17%)
Lower respiratory tract infection	14 (33%)	2 (5%)	7 (17%)	2 (5%)	5 (12%)	12 (29%)
Skin soft tissue infection	11 (31%)	6 (17%)	4 (11%)	2 (6%)	3 (9%)	9 (26%)
Surgical site infection	4 (40%)	2 (20%)	2 (20%)	0 (0%)	0 (0%)	2 (20%)
All	56 (37%)	17 (11%)	22 (15%)	13 (9%)	10 (7%)	33 (22%)

Rank 1: Treatment success with no TEAEs (Best)

Rank 2: Treatment success with TEAEs not leading to discontinuation of eravacycline

Rank 3: Treatment success with TEAEs leading to discontinuation of eravacycline

Rank 4: Treatment failure and no TEAEs

Rank 5: Treatment failure with TEAEs

Rank 6: Death (Worst)
